# Correction to “Development of an Agent‐Based Model to Investigate Durability of Factor IX Activity in Hemophilia B Patients Treated With Etranacogene Dezaparvovec”

**DOI:** 10.1002/psp4.70312

**Published:** 2026-07-27

Li Y , Nandy P , Jordie E , et al., “Development of an Agent‐Based Model to Investigate Durability of Factor IX Activity in Hemophilia B Patients Treated With Etranacogene Dezaparvovec.” CPT: Pharmacometrics and Systems Pharmacology 2026;15(7):e70286. 10.1002/psp4.70286.42322300 PMC13282921

In the first paragraph of the “3.2 Long‐Term Predictions and Drivers of Durability” section, the text “The model‐predicted mean FIX activity at Year 5 was 23.5% (95% CI 4.92%, 72.2%), overlapping with the reported range of 31.6% ± 15.7% (mean ± standard deviation) [31].” was incorrect.

This should have read: “The model‐predicted mean FIX activity at Year 5 was 27.7% (95% CI 4.92%, 72.2%), overlapping with the reported range of 31.6% ± 15.7% (mean ± standard deviation) [31].”

We apologize for this error.

